# Minimum acceptable diet and associated factors among children aged 6–23 months in Ethiopia: a systematic review and meta-analysis

**DOI:** 10.1186/s12887-024-04635-z

**Published:** 2024-02-29

**Authors:** Kedir Abdela Gonete, Dessie Abebaw Angaw, Lemma Derseh Gezie

**Affiliations:** 1 Caring Futures Institute, College of Nursing and Health Sciences, Flinders University, Adelaide, SA Australia; 2 Department of Human Nutrition, Institute of Public Health, College of Medicine and Health Sciences, University of Gondar, P. O. Box 196, Gondar, Ethiopia; 3 Department of Epidemiology and Biostatistics, Institute of Public Health, College of Medicine and Health Sciences, University of Gondar, P. O. Box 196, Gondar, Ethiopia

**Keywords:** Minimum acceptable diet, Meal frequency, Dietary diversity, Systematic review, Ethiopia.

## Abstract

**Background:**

To ensure a child's full growth, health, and development during infancy and the early years, adequate nutrition is crucial. A crucial window of opportunity for ensuring children's proper growth and development through adequate eating exists during the first two years of life. According to the evidence of the efficacy of interventions, achieving universal coverage of optimal breastfeeding could prevent 13% of deaths in children under the age of 5 worldwide, and using complementary feeding methods appropriately would lead to an additional 6% decrease in under-five mortality.

**Methods:**

From several electronic databases, all published, unpublished, and gray literature was extracted and exported into EndNote version X20. For further analysis of the review, the retrieved data from the excel sheet were imported into the statistical software program Stata version. Metanalysis was used to determine the prevalence of MAD, and a random effects model was used to estimate the pooled prevalence of MAD. The DerSimonian-Laird Random effects model (REM) was used to combine the determinant factors from all qualifying papers for the meta-analysis, and the heterogeneity was independently assessed using a χ2 test, Q statistics, and matching I2 statistics. To retrieve the extent of publication bias, funnel plots were scattered and tested for asymmetry and, additionally, Egger’s test was computed with the user-written “meta bias” command in Stata (version 11) software. To end, sensitivity analyses with trim and fill were performed.

**Results:**

The pooled estimate of the overall prevalence of minimum acceptable diet in 16 studies in Ethiopia was 22% with (95% CI: 16, 28%) with a random effect model. However, eight papers were filled during trim and fill in order to counteract the small study effect. The overall filled pooled estimate was 7.9% with (95%CI: 11, 14.8%). Maternal education (primary and secondary) is 1.714 (95% CI 1.244,2.363) and 2.150(95% CI: 1.449,3.190), respectively, Ages of children with range of 12–17 months (2.158 (95% CI 1. 9,3.006) and 18–23 months 2.948(95% CI: 1.675,5.190)), Nutrition information ((1.883 (95% CI 1.169,3.032)) media exposure (1.778(95% CI: 1.396,2.265), and maternal knowledge (2.449 (95% CI 1.232, 5.027) were significantly associated with MAD.

**Conclusion:**

The pooled estimate of the overall prevalence of minimum acceptable diet in 16 studies in Ethiopia were low. Maternal education (primary and secondary), ages of child with range of 12–17 month and 18–23 months, mothers having nutrition information, mothers who have media exposure,and mothers having good knowledge were significantly associated with Minimum acceptable diet. The government, NGO, and other stakeholders should focus on improving Minimum acceptable diet among 6 to 23 months of children through promoting with mass media, focuses on nutrition council during critical contact point in health facility, and doing capacity building for the mothers/caregivers.

## Background

Mothers' and children's health and nutritional status are closely related. In order to improve newborn and young child feeding, it is important to first ensure the health and nutritional status of women as individuals at all phases of life. This is followed by women's roles as parents and caregivers for their families. Mothers and infants form a biological and social unit; they also share problems of malnutrition and ill health. Whatever is done to address these issues affects mothers and children together [1]. Children have a right to appropriate nutrition and access to healthy food, both of which are necessary for them to achieve the best possible level of health [1].

Adequate nutrition during infancy and early childhood is essential to ensure the growth, health, and development of children to their full potential [2–4]. The first two years of life offer a vital window of opportunity for ensuring children's adequate growth and development through optimal feeding [4]. According to the evidence of the effectiveness of interventions, attaining universal coverage of optimal breastfeeding could prevent 13% of deaths in children under the age of 5 worldwide, as well as using complementary feeding methods appropriately would lead to an additional 6% decrease in under-five mortality [5].

The Minimum Acceptable Diet (MAD) for children 6–23 months old, is one of eight core indicators for assessing infant and young child feeding (IYCF) practices developed by the WHO in 2007. The MAD indicator is a composite indicator composed of the Minimum Dietary Diversity (MDD) and Minimum Meal Frequency with the assumption of those who have taking breast feeding and non-breastfeeding [6].

Poor nutrition raises the risk of sickness and is to account for one-third of the estimated 9.5 million deaths of children under the age of five that occurred in 2006 [7, 8]. Forty five percent(45%) of child mortality are attributed to undernutrition. In 2020, it was predicted that 45 million children under the age of 5 were wasted (too little for their height), and that 149 million children under the age of 5 were stunted (too short for their age). These conditions were caused by insufficient dietary intake [9]. On the other side, the Asia and Pacific region had an estimated 31.5 million wasting people and 74.5 million stunted children under the age of five. The majority of these children in the region live in Southern Asia with 55.9 million stunted and 25.2 million wasted children [10]. In India, children aged 6 to 23 months consume a minimum acceptable diet (MAD) to the extent of 4 to 9%, while in the Philippines, just 6.7% of children meet the requirements for a minimum acceptable diet [11, 12]. Studies carried out in Indonesia and Bangladesh revealed that 20 and 40% of children get MAD, respectively [10, 13].

The highest prevalence of undernutrition have been seen in the African region and South-East Asia, where the former is responsible for about 39.4% of stunted, 24.9% of underweight, and 10.3% of wasted children under the age of five [14]. According to the 2015 Millennium Development Goals (MDG) report, sub-Saharan Africa (SSA) is responsible for one-third of all undernourished children worldwide, emphasizing the fact that malnutrition is still a significant health issue for children under the age of five in the sub-region [15]. According to the data from the Demography and Health Survey, children in Sub-Saharan African countries between the ages of 6 and 23 months had an overall prevalence of the minimum acceptable diet consumption of 9.87%, ranging from 3.10% in Guinea to 20.40% in Kenya. The findings show that Sub-Saharan African children aged 6 to 23 months have better access to meal frequency than dietary diversity [16]. According to the Ethiopian Demographic Health Survey, the percentage of infants and young children in Ethiopia who consumed the Minimum Acceptable Diet during the survey periods of 2011, 2016, and 2019 was 4.1%, 7.3%, and 11.3%, respectively [17].

Poor eating habits affected growing children in several ways, including lowering immunity and the body's capacity to fight against illnesses, reducing physical fitness, and increasing the risk of several deficiency conditions like anemia from a lack of iron and osteoporosis from a lack of calcium [18, 19]. Furthermore, a poor diet puts children at risk for impaired learning, increased infections, poor brain development, and, in many cases, mortality [20].

Poor infant and young child feeding practices, in particular MAD, have a variety of major causes, including sociodemographic and economic factors such as having a husband with a secondary or higher educational level [21–23], being a housewife [21], having mothers early ages [17, 21], child late ages [21–25], having households with rich and middle-class wealth [17, 24, 26], having mothers who are married [24], mothers who are educated [17, 22, 25–27], involvement of mother in decision making [27], birth order of index children [27], and employed mothers [23]. Having a history of postnatal follow-ups [17, 21, 28], institutional delivery [25–27], using Growth Monitoring Promotion services [22, 25, 28], having Antenatal Care visits [22], and having a history of illness two weeks prior to the survey [22] are characteristics related to child and mother health care utilization.. Besides, in maternal/caregiver knowledge characteristics of children like media exposure [17, 23, 25, 26, 28], having good knowledge about child feeding practice [27, 28]. Furthermore, living in the household with home garden [22] are significantly associated with MAD.

Both UNICEF and WHO had produced infant and young child feeding guidelines and interventions for children [29]. Ethiopia also employed this IYCF technique for mothers and their young children. Over the past twenty years, the Ethiopian government (GoE) has developed and put into action a number of pro-poor policies and programs that have been centered on food security, mother and child health, and multisectoral poverty reduction. The government of Ethiopia has been designing NNPI, NNPII, and food and nutrition policies for the past 20 years with a focus on mother and child health practice and intervention. Such a strategy is also supported by several government and non-government planned strategies [30, 31]. For instance, the Government of Ethiopia exemplified its commitment to nutrition by issuing the Seqota Declaration to eliminate child undernutrition and end stunting in Ethiopia by 2030. The 15-year Seqota Declaration implementation Plan focuses on delivering high-impact nutrition-specific, nutrition-sensitive, and infrastructure interventions across multiple sectors [32] Stunting rates have steadily decreased over the past 20 years, but in 2019 they were still high—38% for children under five and 28% for those under two (with significant regional variation). In all parts of Ethiopia, there is still a serious public health issue. In Ethiopia, children still receive a poor or insufficient amount of a varied food. According to EDHS 2016, the prevalence of Minimum acceptable diet among 6–23 is not more than 7percent. The aforementioned measurement as a whole demonstrated that poor MAD is a key aspect for Ethiopian children and a national public health concern. Therefore, the goal of this systematic review and meta-analysis is to assess the pooled prevalence and contributing variables of the Minimum Acceptable Diet among children aged 6–23 months in Ethiopia.

## Methods

### Design and searching strategy

This systematic review and meta-analysis were done to organize the most updated and recent published articles to enhance pooled evidences data on the prevalence and associated factors of minimum acceptable diet among 6 to 23 months of children in Ethiopia. To organize the Systematic Review and Meta analysis report, we used the protocol of the Preferred Reporting Items for Systematic Review and Meta-Analysis (PRISMA 2020 Statement) guideline [33].

Various search engines were used to look up significant publications in the databases of PubMed, HINARI, advanced Google Scholar, Web Science, EMBASE, SCOPUS, Epistemonikos, science direct, and free Google. Additionally, despite the fact that EMBASE, SCOPUS, and Web Science charge a money for each published paper, they have strict guidelines that allow individuals with institutional email addresses to apply for a free pay chance. As a result, to search the published paper on this database, we utilized our institutional email. Three authors conducted separate searches and reviews of all the publications, both published and unpublished: (KAG), (DAA), and (LDG). The following search terms were used to find articles on December 25–26, 2022: "Minimum Acceptable Diet" [Title/Abstract] AND ("children's" [Title/Abstract] OR "Child" [Title/Abstract] OR "Minors" [Title/Abstract] OR "Only Child" [Title/Abstract] OR "6 to 23 months" [Title/Abstract]))). For the other databases, we employed specific-subject headers (copied and pasted the themes) (HINARI and advanced Google Scholar). In addition, we manually scanned the reference lists of accepted publications to find more appropriate literature.

### Definition of concepts

Complimentary food (CF): is any solid, semi-solid, or soft food, whether manufactured or locally prepared, suitable as a complement to breast milk or infant formula to satisfy the nutrient requirement [34]. While minimum dietary diversity (MDD): those children 6–23 months of age who receive foods from four or more food groups during the previous day considered to have adequate MDD. The seven food groups used for tabulation of this indicator were: grains, roots, and tubers; legumes and nuts; dairy products (milk, yogurt, and cheese); fresh foods (meat, fsh, poultry, and liver/organ meats); eggs; vitamin A-rich fruits and vegetables; and other fruits and vegetables [35]. Minimum meal frequency (MMF) are those children both breastfed and non-breastfed children 6–23 months of age who receive solid, semi-solid, or soft foods the minimum number of times or more in the previous day was considered as having adequate MMF [27]. But minimum acceptable diet (MAD) is considered as adequate if children’s received the minimum acceptable diet (both minimum dietary diversity and minimum meal frequency) during the previous 24 h for both breast milk and non-breast milk [27]. HFIAS (household food insecurity access scale):—was assessed from FANTA (Food and Nutrition Technical Assistance) 2007 with nine main questions, HFIAS divided into food secure if the summations were ≤ 1 point out of 27 scores while the household food security level of the summations ≥ 2 points out of 27 scores were considered as food insecure [36]. Whereas Wealth index is a composite measure of a household’s cumulative living standard places individual households on a continuous scale of relative wealth tertile (rich, middle, and poor) [37]. At last, nutrition information/Media Exposure are those who have exposed to either of the following medias like TV, Radio for nutrition information [26].

### Selection of studies

From several electronic databases, all published, unpublished, and gray literatures were extracted and exported into EndNote version X20. The three independent reviewers (KAG, DAA, and LDG) carefully examined the titles, abstracts, and full texts of the papers in order to screen out any duplicate articles on the endnote. Further conversation among the reviewers helped to settle a disagreement between them.

### Eligibility criteria

#### Inclusion criteria

### Setting/context

This systematic review and meta-analysis were contained within the study done on Ethiopia. Unfortunately, only the published paper included all of the extracted findings.

### Population and condition

All mothers who have children between the ages of 6 and 23 months were part of the study and were recognized as a mother–child pair.

### Study design

The review centered on an observational (cross-sectional study) that sought to determine the prevalence of a minimum acceptable diet and/or the associated factors among Ethiopian children aged 6 to 23 months.

### Language

Studies that were conducted in English were included.

### Outcome

The prevalence of Minimum acceptable diet is considered as the outcome variables. The outcomes were calculated using the minimum amount of dietary diversity and meal frequency, assuming that the children are currently or have ever been breastfed.

### Publication year

All studies that were released before December 25, 2022.

### Exclusion criteria

Studies that did not account for all essential information on the outcome and predictors were rejected. Additionally, the reviews did not include research done for case reports, case series, letters to the editors, or correspondence.

### Data extraction and management

Three authors (KAG, DAA, and LDG) summarized the studies conducted on the prevalence and associated factors of the Minimum Acceptable Diet among children aged 6 to 23 months in Ethiopia using the data extraction format that was created with the help of the Joanna Briggs Institute (JBI) data extraction tool for prevalence studies. The extracted data were then compared between the three authors (KAG, DAA, LDG). With further conversation between the reviewers—sometimes with the third reviewer acting as a mediator for further debate—differences between the two or more were resolved. For each study, the first author's name, the study's location, the area where the papers were published, the year of publication, the study's design, sample size, the prevalence of the minimum acceptable diet, or the number of events or cases having an adequate MAD with the associated factor (age of mothers and children, ANC, PNC, household food insecurity, education levels, wealth index, current breastfeeding, nutrition information, decision-making, media exposure, and knowledge about IYCF and residency) with their standard error were extracted.

### Risk of bias and quality assessment

The three reviewer authors (KAG, DAA.LDG) independently assessed the qualities of the eligible studies and controlled for possible bias by using protocol and the criteria proposed in the Newcastle–Ottawa Scale customized for cross-sectional studies [38]. The two reviewers' discrepancies were minimized during the quality assessment by exchanging thoughts with one another. In the same way, the third reviewers bargain their opinions in order to facilitate additional dialogue.

### Statistical analysis

The data from the generated excel sheet were imported into Stata version statistical program for additional review analysis. The ratio of events (the number of appropriate MAD participants)/total (the total number of study participants) and metanalysis were used to establish the prevalence of the Minimum Acceptable Diet. Random effects were used to assess the pooled prevalence of MAD. By examining the adjusted ORs and 95% CIs given in each study, several determining factors for MAD that satisfied the meta-analysis inclusion requirements were found. A random effects model was used to pool the determinant factors taking into account both the sampling error and between study heterogeneity by the generic inverse variance method by the user written “metan” command [39]. Determinant factors were pooled from all eligible studies using the DerSimonian-Laird Random effects model (REM) for meta-analysis [40]. The heterogeneity was separately estimated using a χ2 test, Q statistics with corresponding I2 statistics. The heterogeneity was alleviated by doing sub-group analyses for MAD of region category in Ethiopia. Prevalence rates, odds ratios (OR), and 95% confidence intervals (CI) for each study included in the model as well as for the overall estimate were calculated and shown in tables and forest plots. Funnel plots were dispersed, evaluated for asymmetry, and Egger's test was computed using the user-written "meta bias" command in Stata (version 11) software to determine the degree of publication bias [39]. Finally, trim and fill sensitivity analyses were carried out [41].

## Results

### Description of included studies

**Fig. 1:**
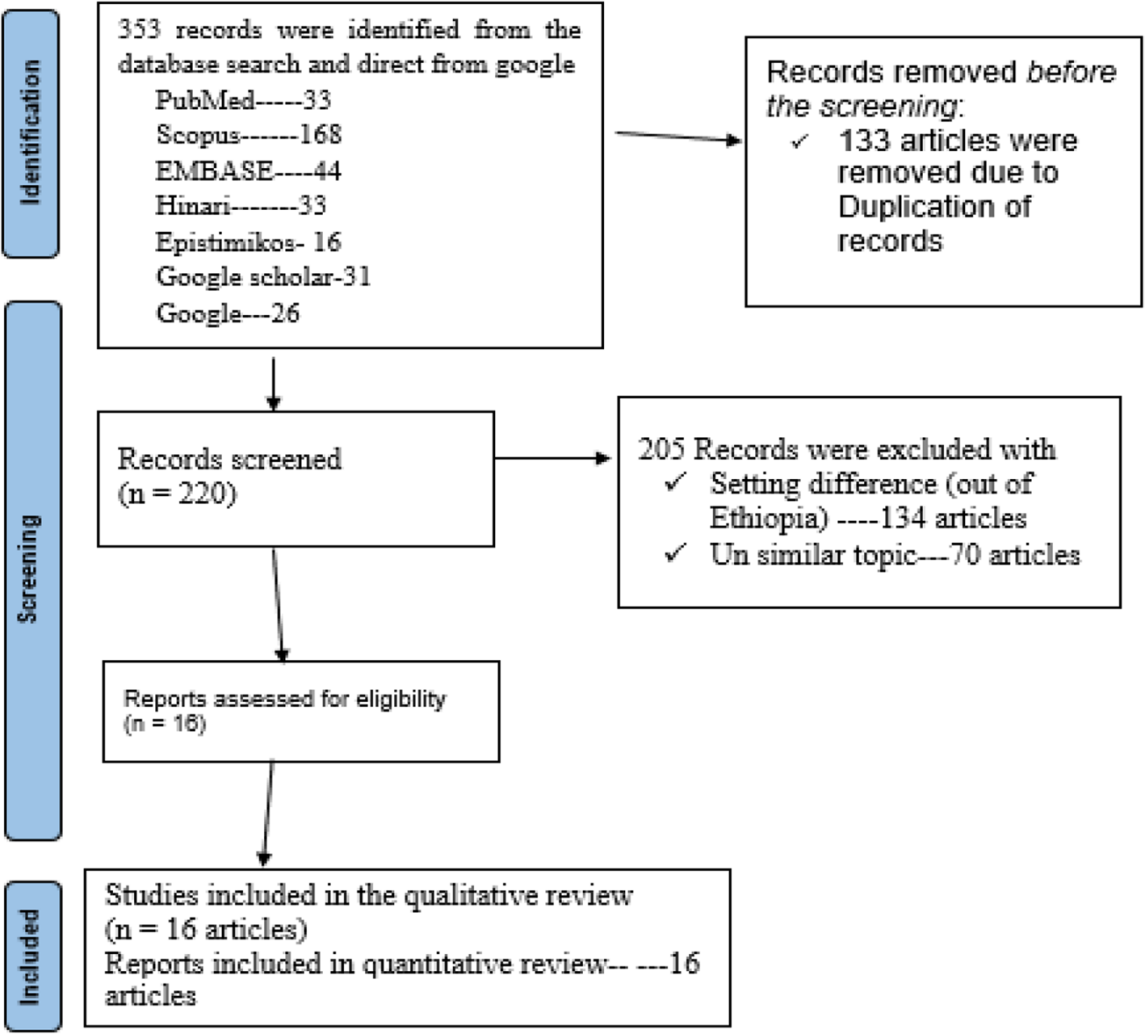
A PRISMA flow chart illustrating the study selection process included for systematic review and meta-analysis Minimum acceptable diet among 6 to 23 months in Ethiopia

We searched 353 published publications on various electronic databases from international peer-reviewed journals. 133 papers/articles were left out as a result of duplication. The independent reviewers began their screening process by looking at the titles and abstracts of 220 papers. They discovered 134 publications were not from Ethiopia, and another 70 papers did not adhere to the topics. Finally, 16 papers—both qualitative and quantitative—were included in the most recent review (Fig. 1). Six papers from the sixteen studies were discovered in Amhara state, while four more were discovered in the Oromia region. Ethiopian nationals from Addis Ababa and the South each have one article. However, four studies using big data analysis from the Ethiopian Demographic Health Survey were published covering every area of Ethiopia. The final analysis included a sample size of 14,045 individuals (Table 1).

**Table 1:** Characteristics of the included studies and prevalence of Minimum acceptable diet in individual studies

S/N	author	year of published	study region	design	Sample size/total population	Event/disease	Proportion
1	Abebe.et.al	2021	Addis Abeba	cross sectional	562	419	0.7455516
2	Hareg/Birehan et.al	2022	Amhara	cross sectional	738	143	0.1937669
3	Birie et.al	2021	Amhara	cross sectional	430	54	0.1255814
4	Feleke et.al	2020	South Ethiopia	cross sectional	662	235	0.3549849
5	Gizaw et.al	2019	Oromia	cross sectional	200	27	0.135
6	Molla et.al	2020	Amhara	cross sectional	531	168	0.3163842
7	Mulat et.al	2019	Amhara	cross sectional	502	49	0.0976096
8	Tasew et.al	2019	All Region/Ethiopia	cross sectional	2919	178	0.0609798
9	Teshome et.al	2022	All Region/Ethiopia	cross sectional	1457	165	0.1132464
10	Tsedal et.al	2020	All Region/Ethiopia	cross sectional	1307	105	0.0803366
11	Ahmed et.al	2022	Oromia	cross sectional	536	224	0.4179104
12	Kedir Yimam et.al	2021	All Region/Ethiopia	cross sectional	2864	203	0.0708799
13	Getahun Ersino et.al	2016	Oromia	cross sectional	413	25	0.0605327
14	Getahun Ersino et.al	2016	Oromia	cross sectional	217	20	0.0921659
15	Esubalew et.al	2018	Amhara	cross sectional	420	155	0.3690476
16	Hiwot Yisak et.al	2020	Amhara	cross sectional	287	100	0.3484321

### The pooled estimate of minimum acceptable diet among 6 to 23 months in Ethiopia

**Fig. 2:**
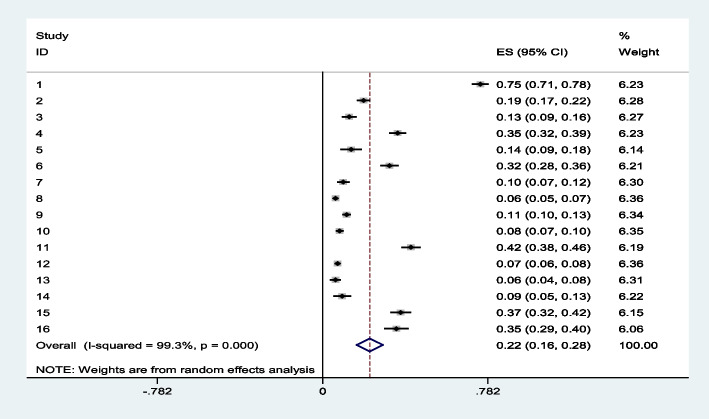
forest plot for meta-analysis of minimum acceptable diet among children in 6 to 23 months in Ethiopia

**Fig. 3:**
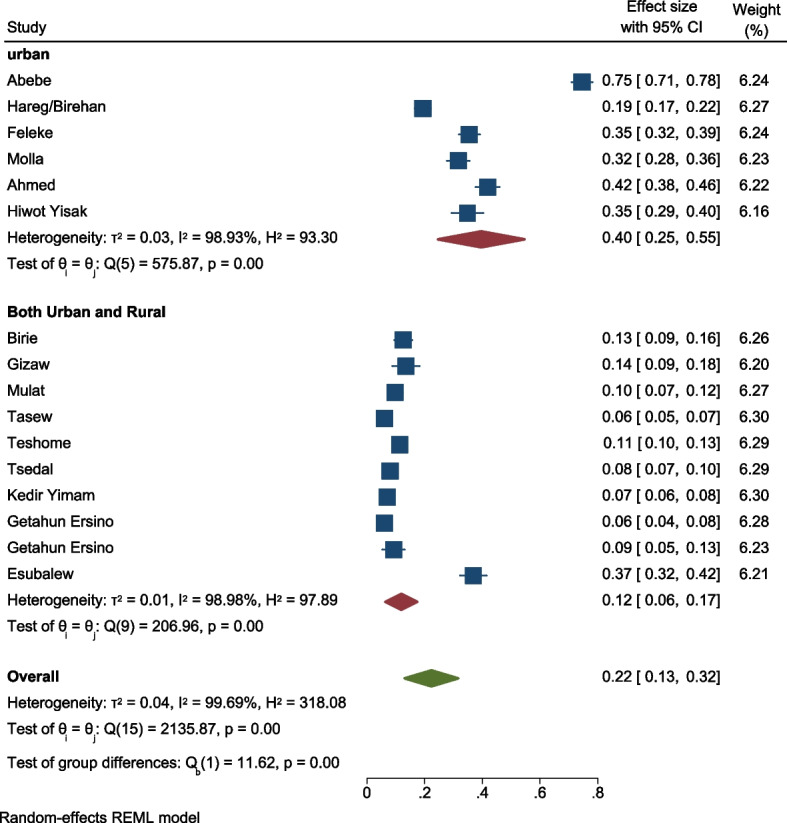
Forest plot for meta-analysis of minimum acceptable diet among children in 6 to 23 months in subgroup analysis with residence

The pooled estimate of the overall prevalence of minimum acceptable diet in 16 studies in Ethiopia were 22% with (95% CI: 16, 28%) and possible heterogeneity was observed in the studies (I^2^ = 99.3, *p* value = 0.001) (Fig. 2). But with considering residence difference, we did subgroup analysis of pooled estimate of MAD. Among the 16 reviewed papers, six of them were done in Urban residence. In Urban residence, the overall pooled prevalence of MAD was 40% with (95%CI: 23,56% I^2^ = 98.93, *P*value = 0.001) whereas, the rest of the ten papers were done in both Urban and rural. Both urban and rural residences are much lower than Urban with estimated pooled prevalence of 11% (95%CI: 9,14% I^2^ = 98.98 *P*value = 0.001) (Fig. 3). The pooled effect of the proportion of MAD in all the two category of sub group analysis did heterogeneity.

### Handling heterogeneity

**Fig. 4:**
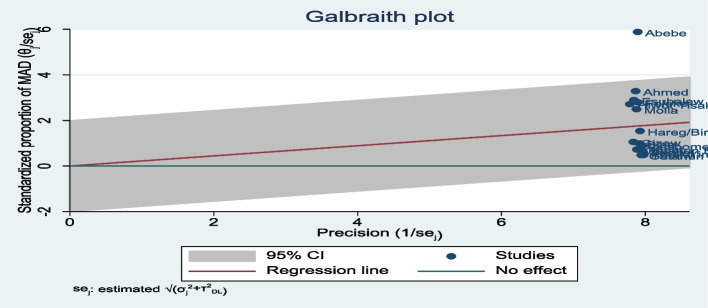
Galbraith graph for MAD heterogeneity

**Fig. 5:**
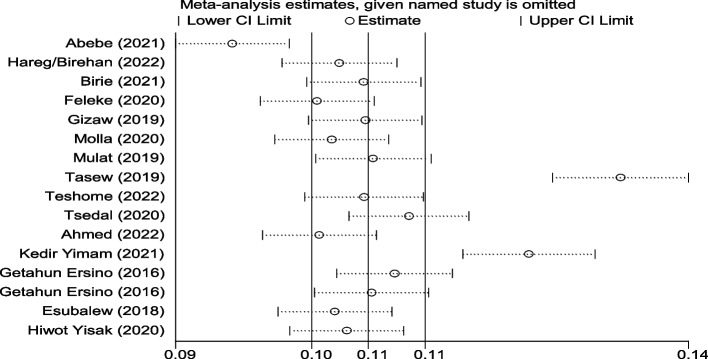
Sensitivity analysis for MAD heterogeneity with fixed effect model

**Fig. 6:**
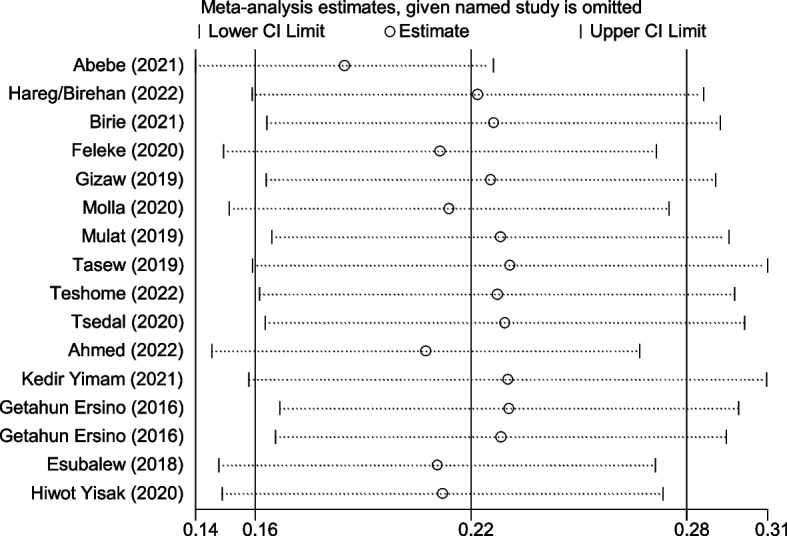
Sensitivity analysis for MAD heterogeneity with random effect model

The degree of heterogeneity among the included studies was high in the pooled estimation of the random effects model. Heterogeneity was verified at the statistical level (I2 = 99.3%) as well as at the visibility level (forest plot and Galbraith) (Fig. 4). In the Galbraith graph, one paper was obtained out of 95% CI and it showed the presence of heterogeneity. To do so, sensitivity, subgroup, and meta-regression analyses were performed. The sub-group analysis was conducted taking into consideration the place of residence of the 16 studies. However, there was a high degree of heterogeneity between the two categories of residence (Fig. 3). Similarly, Sensitivity analysis with a fixed effect model showed, three papers were presented as influential and out of the range of the confidence interval (Fig. 5), but by adding random on the meta commend, all the papers were under the confidence interval (Fig. 6) and did not have any influential study.

### Meta regression

To manage heterogeneity, we also adjusted the Meta regression on the aggregated variables at the study level using the randomized effect model. Meta regression analysis found that residence was significantly associated with a minimal acceptable diet (Table 3). Based on the results in Table 3, the statistical analysis (I2 = 98.21%) showed strong heterogeneity between residents. Furthermore, depending on the *p*-value (0.001), it is significantly associated with the MAD. As a result, the source of heterogeneity was deference towards residence (Table 2).

**Table 2:** Meta-regression analysis results for the prevalence of Minimum acceptable diet among 6 to 23 months in Ethiopia

Proportion	Exp(B)	Std. Err	t	*P* > (t)	Num. observation	I-square	95% Confi. Interval
Residence	.8709701	.029945	4.02	0.001	16	98.21%	0.8090554, 0.937623

### Factors/barriers to minimum acceptable diet

Sixteen studies were included to estimate the overall pooled effect size of different factors reported repeatedly that affect the minimum acceptable diet age 6–23 months in Ethiopia. Accordingly, twelve risk factors (maternal [21, 42] and child age [22, 42, 43], ANC [21, 22], PNC [21, 24, 25, 28, 44], Household food insecurity [21, 22, 42], maternal education [21, 25, 45], wealth index [22, 25, 28, 42–44], nutrition information [27, 28, 42], mothers’ decision power [26, 27], media exposure [23, 25, 27, 43, 46], maternal knowledge [26, 47] and residence [17, 28, 48]) had data that could be used in the quantitative meta-analysis. Greater heterogeneity was observed among studies evaluating maternal decision-making, the age of the child, and being both middle and rich wealth groups (Table 3). On the pooled estimate of odd ratio, maternal education (primary and secondary), age of the child (18–23 months), nutrition information, media exposure, and maternal knowledge is significantly associated with minimum acceptable diet.

**Table 3:** Pooled estimate of factors associated with MAD among children 6 to 23 months in Ethiopia

Variable name	Variable Category	Number of studies	Pooled estimate (OR)	Heterogeneity chi-squared (*P* value)	95% confi. Interval	I^2^ -Square (Heterogeneity)
Age of Mother	Age of mother from 25–34	2	1.658	0.207	0.973, 2.826	37.3%
	Age of mother from 35–49	2	1.792	0.999	0.258,17.442	0.00%
ANC	Antenatal care > _4 visit	2	1.425	0.162	0.733,2.769	48.8%
PNC	Having PNC	5	1.562	0.064	0.956, 2.360	54.9%
House hold food security	House hold food security_secured	3	1.328	0.697	0.983, 1.794	0.00%
Maternal Education	Mother educational status_primary	3	1.714	0.477	1.244, 2.363	0.00%
	Mother educational status_secondary and above	3	2.150	0.932	1.449, 3.190	0.00%
Wealth Index	Wealth index_middle	6	1.548	0.010	0.868, 2.762	66.9%
	Wealth index_rich	6	1.929	0.001	0.962, 3.871	79.6%
Age of Child	Age of child_12-17 month	3	2.158	0.671	1.549,3.006	0.00%
	Age of child_18-23 month	3	2.948	0.055	1.675, 5.190	65.6%
Nutrition information	Nutrition information_yes	3	1.883	0.366	1.169, 3.032	0.6%
Mothers Desicion making	Desicion making _Mother involved	2	2.322	0.015	0.630,8.553	83%
Media Exposure	Media Exposure_satisfactory	5	1.778	0.338	1.396, 2.265	11.91
Maternal Knowledge	Maternal Knowledge about IYCF_good	2	2.449	0.197	1.232, 5.027	39.9%
Residence	Residence_Rural	3	1.310	0.227	0.443,3.880	32.6%

**Fig. 7:**
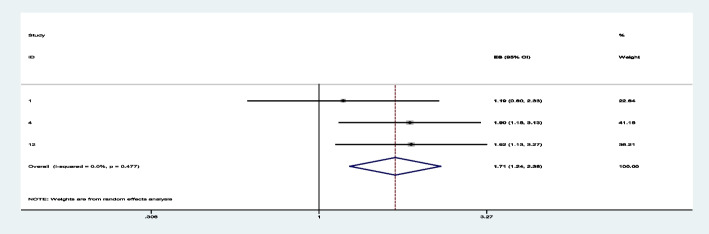
The overall pooled odds ratio of the association between maternal education (being primary) and the MAD among children aged 6 to 23 months in Ethiopia

**Fig. 8:**
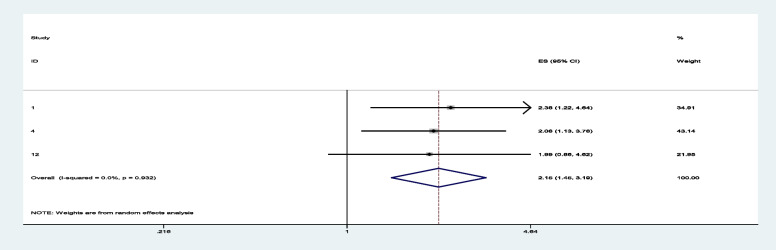
The overall pooled odds ratio of the association between maternal education (being secondary and above) and the MAD among children aged 6 to 23 months in Ethiopia

**Fig. 9:**
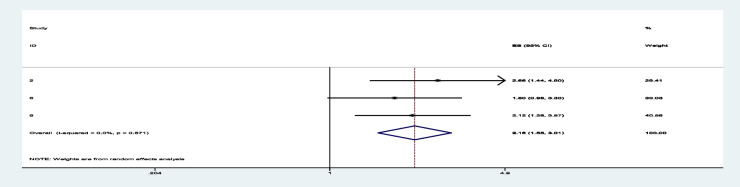
The overall pooled odds ratio of the association between age of child (12-17 months) and the MAD among children aged 6 to 23 months in Ethiopia

**Fig. 10:**
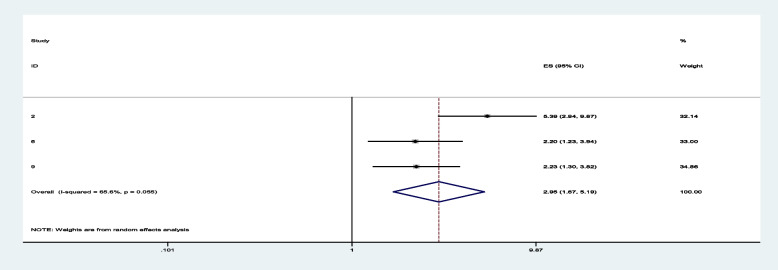
The overall pooled odds ratio of the association between age of child (18-23 months) and the MAD among children aged 6 to 23 months in Ethiopia

**Fig. 11:**
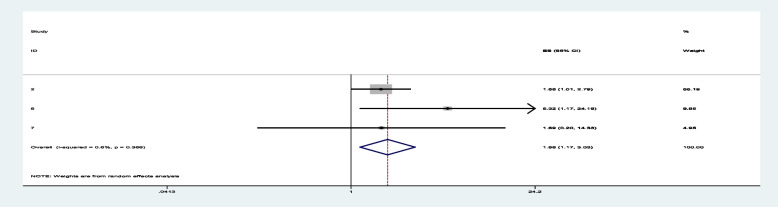
The overall pooled odds ratio of the association between having nutrition information and the MAD among children aged 6 to 23 months in Ethiopia

**Fig. 12:**
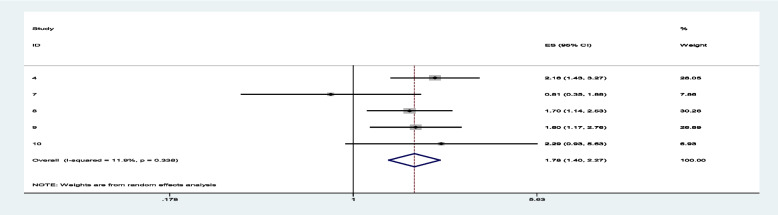
The overall pooled odds ratio of the association between having media exposure and the MAD among children aged 6 to 23 months in Ethiopia

**Fig. 13:**
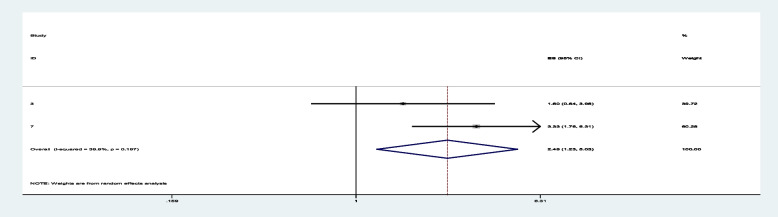
The overall pooled odds ratio of the association between maternal knowledge and the MAD among children aged 6 to 23 months in Ethiopia

Among sixteen studies, three of them revealed that maternal education (primary and secondary) (Figs. 7 and 8) was significantly associated with minimum acceptable diet among children aged 6– 23 months, with the odds ratio of 1.714 (95% CI 1.244,2.363) and 2.150(95% CI: 1.449,3.190), respectively (Table 3). This indicates that, the odds of having good minimum acceptable diet were 1.714 and 2.150 times (1.714 (95% CI 1.244,2.363) and 2.150(95% CI: 1.449,3.190)) higher among mothers with having elementary and secondary and above education level compared to those illiterate mothers, respectively. Similarly, the odds of having MAD were 2.158 and 2.948 times (2.158 (95% CI 1.549,3.006) and 2.948(95% CI: 1.675,5.190)) higher among ages of child with range of 12–17 month and 18–23 months compared to those age range of 6–11 months of child (Figs. 9 and 10). In addition, the odds of having MAD were 1.883 times ((1.883 (95% CI 1.169,3.032)) higher among mothers who have nutrition information compared to did not have nutrition information (Fig. 11). Likewise, the odds of having MAD were 1.778 times (1.778(95% CI: 1.396,2.265) higher among mothers who have media exposure compared with counter parts (Fig. 12). Finally, the odds of having MAD were 2.449 times (2.449 (95% CI 1.232, 5.027) (Fig. 13) higher among mothers having good knowledge compared to the counter (Table 3).

### Publication bias

**Fig. 14:**
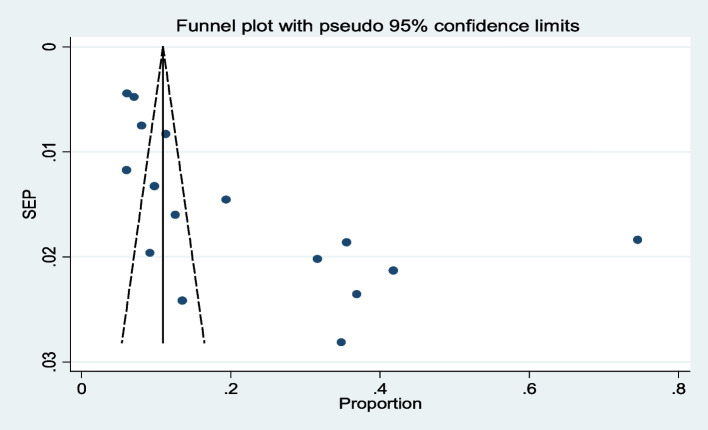
Funnel Plot to Assess Publication Bias for MAD

**Fig. 15:**
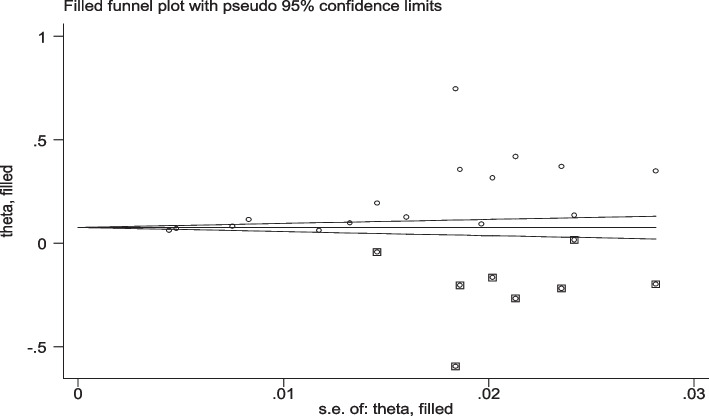
Trim and fill for small study effect of MAD

Publication bias was assessed using the funnel graph for symmetry by visual inspection of the minimum acceptable diet and appeared quite asymmetrical and revealed publication bias (Fig. 14). Egger's test also showed a publication bias [Egger's test *p* = 0.003] (Table 4). This visual and statistical test showed that there is a small study effect in the review and that the problem of bias has been attenuated with trimming and fill (Fig. 15). During trimming and fill, eight papers were filled to adjust the small study effect. The random effect model of the overall filled pooled estimate were 7.9% with (95%CI: 11, 14.8%) (Table 5).

**Table 4:** Egger’s test for publication bias

S td_Eff	Coef	Std. Err	t	*P* >|t|	[95% Conf. Interval]
Slope	**-.0087794**	**.0390252**	**-0.22**	**0.825**	**-.0924801, .0749212**
Bias	**14.48605**	**3.97684**	**3.64**	**.003**	**5.956575, 23.01552**

**Table 5:** Pooled estimate of the filled, meta-analysis trim and fill

Method	Pooled estimate	95%CI		Asymptotic		No of studies
		**LOWER**	**UPPER**	**Z-value**	***P*-value**	
**Fixed**	**0.075**	**0.071**	**0.080**	**32.425**	**0.000**	**24**
**Random**	**0.079**	**0.011**	**0.148**	**2.267**	**0.023**	

Test for heterogeneity: Q = 4612.544 on 23 degrees of freedom (*p* = 0.000)

Moment-based estimate of between studies variance = 0.029

## Discussion

Adequate nutrition for children is essential to the achievement of their full developmental potential. Under-nutrition has crucial implications for physical and cognitive growth and development. Malnutrition leads to failure in early physical growth, retarded motor competence, and cognitive and behavioural development. Children who continue to suffer malnutrition in early childhood have difficulties compared to those who have adequate nutrition and a healthy living environment [49, 50]. The aim of this review is to assess the pooled estimate of Minimum acceptable diet and associated factors among 6 to 23 months in Ethiopia.

The pooled estimate of the overall prevalence of the minimum acceptable diet across 16 studies in Ethiopia was 22% (95% CI: 16. 28%). Despite searching different databases, we were unable to find systematic reviews or meta-analyses on the minimum acceptable diet and factors between 6 and 23 months. Therefore, the papers reviewed were discussed with those of the national studies. The pooled estimate of our review is in line with the 2011 DHS study in Nepal with 26.5% [51]. But, the pooled estimate from our review is higher than the analysis of national health and demographic data from India [52] and Pakistan [53]. The survey may have revealed a discrepancy because two-thirds of Indian households (65.5%) were considered poor, which may have affected the quality and quantity of dietary diversity, MAD, meal frequency, accessibility, and availability of household members to children's pairs. In addition, most of the study subjects were from districts with high and low burden. The Pakistani study found that the majority (83.5 per cent) of mothers and parents had no form of employment and this affects children's access to food. Similarly, our study outperforms studies conducted on subSaharan and East African studies demographic and health survey data. The pooled prevalence of MAD was 9.89% and 11.56% in subSaharan and East Africa, respectively. This difference may be due to the large number of countries participating in both studies. This country had inconsistent prevalence (higher in some, very low in others). Therefore, prevalence decreases during pooled estimation. In addition, there are differences in socioeconomic characteristics [16, 54].

Likewise, the pooled estimate of this review was higher than the study on Ethiopia. According to the EDHS 2016 results, the feeding practices of children in Ethiopia aged 6–23 months were 7%. This could be due to the fact that the EDHS is carried out on a national level throughout the region. Although the 16 items reviewed were addressed to the larger regions, some smaller regions did not find primary study in the review. Additionally, EDHS data were also used large sample size in all thematic areas in the regions [55]. According to the systematic review on complementary feeding practice among 6 to 23 months in Ethiopia, the pooled prevalence was only 10%. This difference might be due to the study were addressed the high number of reviewed, high sample size, different cutting point or standard of the quality assessment were used [56].

Our pooled prevalence of MAD was also lower than the study done on DHS 2019 [57] Nepalese study with estimated prevalence of 30.1%. The difference might be due to more than three- fourth of the participants were being urban and have accessibility to variety of foods. Likewise, more than half of the mother and more than three-fourth of the father participants enrolled on higher education. Also, nearest to half of the participants also put on rich tertile segment. children in wealthier households grow better for a number of reasons, among which improved nutrient adequacy may be one important mechanism that household wealth and resources translate into better outcomes for children [58, 59]. Wealthier households are expected to have the resources to purchase more food and thus have diverse diets compared to poor households. A higher parental educational level is associated with better employment opportunities and higher incomes, and may translate into higher purchasing power and better nutrition knowledge [58, 59]. Correspondingly, our review also lower estimated prevalence than the study done on DHS 2008 Ghana. The alteration might be due to socio economic, cultural and sample size difference.

After doing trim and fil, the total number of articles were 24 with filled eight papers. The estimated filled pooled prevalence of MAD was 7.9% and 7.5% with random and fixed effect model, respectively. The filled meta-analysis of the pooled estimate is much lower than before filling the meta-analysis (22%). This showed that, the reviewed articles were higher publication bias due to missing the high number of small studies.

The odds of having good minimum acceptable diet were 1.714 and 2.150 times higher among mothers with having elementary and secondary and above education level compared to those illiterate mothers, respectively. This are supported with the study done on the Demographic and health survey data of Nepal(DHS 2011 and 2019) [51, 57], Sub-Saharan Africa [16], East Africa [54], Ethiopia [56], and EDHS 2016 [48]. This might be due to the fact that as the mothers educational level increases, her knowledge, attitude, and practice towards dietary feeding practice might be improved. Majority of their economy also invested to bought variety of healthy food to the children’s. In addition, as the mother’s educational level increases, the level of antenatal care follow-up, Postnatal care, nutrition information, and media exposure will be enhanced with improving variety of food to children’s. Besides to that, having higher education will boost nutritional counseling and improve her child feeding practice [60–62].

Similarly, the odds of having MAD were 2.158 and 2.948 times higher among ages of child with range of 12–17 month and 18–23 months, respectively compared to those age range of 6–11 months of child. This finding is consistent with the study done on DHS (2019)Nepal [57], DHS(2015/16)India [52], and East Africa [61]. This might be due to, good nutrition during the first 2 years of life is vital for healthy growth and development. Starting good nutrition practices early can help children develop healthy dietary patterns. As the age increases, the variety, density and quality of the food must increase and will attain appropriate weight, height/length or other body composition to the children. Also, giving adequate and healthy food to the late age children will make smooth growth and development of children in different body. In addition, the community members believe that giving solid foods from animal sources to children under 12 months of age is inappropriate until their teeth have fully exploded [63]. So, concerns have been raised regarding the improvement of MAD in children aged 6 to 12 months, as well as the timely introduction of complementary foods [64, 65].

In addition, the odds of having MAD were 1.883 times ((1.883 (95% CI 1.169,3.032)) higher among mothers who have nutrition information compared to did not have nutrition information. Those Mothers who have good nutrition information will enhance the probability of giving variety of the food to their child. Also, having good nutrition information will create good opportunity to having full ANC, Nutrition counselling, full PNC and GMP practice. Such, opportunity will increase infant and young children feeding habit [66, 67]. Likewise, the odds of having MAD were 1.778 times (1.778(95% CI: 1.396,2.265) higher among mothers who have media exposure compared with counter parts. This review is consistent with the study done on DHS Sub-Saharan [16], DHS East Africa [61], and DHS India [52], DHS(2019)Nepal [57]. Mass media exposure will increase chance of being good knowledge related to dietary diversity (IYCF). They have also ability to identify the healthy food delivered to children. In addition, having exposure of mass media will enhance access of the health facility during pregnancy and lactating time. So, it increases frequency of ANC, PNC, nutrition counseling during ANC, visiting health facility during child sick, take service of GMP and immunization. Likewise, promotion of nutrition information will also be accessed with watching TV, listening radio, reading magazine, leaf late and newspaper. The above service will boost chance of accessing adequate dietary diversity (MAD) to children [68, 69]. Finally, the odds of having MAD were 2.449 times higher among mothers having good knowledge compared to the counter. Good maternal knowledge through nutrition counseling and education can lead to improved IYCF practices, and consequently, improved child growth and development. Having good knowledge will enhance the proportion of timely initiations of breast feeding, exclusive breastfeeding, complementary breastfeeding and taking colostrum. Such good habit will improve the variety of food and nutritional status to children [70–72].

### Limitations of the study

Even though we are doing sensitivity, subgroup, and meta-regression analyses to minimize the effect of heterogeneity, the degree of heterogeneity among the reviewed articles were high. This might be due to the difference in the study area, study period, residence and other unspecified variations. In addition, strong publication bias also observed on the review due to small study effect. Therefore, researcher and policymakers should consider effects of heterogeneity and result of filled meta-analysis during the interpretations of results.

## Conclusion

Based on the WHO and other guideline recommendation, the pooled estimate of the overall prevalence of minimum acceptable diet in 16 studies in Ethiopia were low. Maternal education (primary and secondary), ages of child with range of 12–17 month and 18–23 months, mothers having nutrition information, mothers who have media exposure,and mothers having good knowledge were significantly associated with Minimum acceptable diet. The government, NGO, and other stakeholders should focus on improving Minimum acceptable diet among 6 to 23 months of children through promoting with mass media, focuses on nutrition council during critical contact point in health facility, and doing capacity building for the mothers/caregivers.

## Data Availability

All data generated or analyzed during this study are included in this published article.

## Abbreviations

DHS: Demographic and Health survey
EDHS: Ethiopian Demographic and Health Survey
GoE: Government of Ethiopia
MAD: Minimum Acceptable Diet
MDD: Minimum Dietary Diversity
MDG: Millennium Development Goal
MMF: Minimum Meal Frequency
NNPI: National Nutrition Program I
NNPII: National Nutrition Program II
PRISMA: Preferred reporting items for Systematic review and Meta analysis
SSA: Sub Saharan Africa
WHO: World Health Organization
